# Determination of Neonicotinoid Pesticides in Propolis with Liquid Chromatography Coupled to Tandem Mass Spectrometry

**DOI:** 10.3390/molecules25245870

**Published:** 2020-12-11

**Authors:** Rok Tomšič, David Heath, Ester Heath, Jernej Markelj, Andreja Kandolf Borovšak, Helena Prosen

**Affiliations:** 1 Faculty of Chemistry and Chemical Technology, University of Ljubljana, 1000 Ljubljana, Slovenia; rok.tomsic16@gmail.com (R.T.); Jernej.Markelj@fkkt.uni-lj.si (J.M.); 2 Department of Environmental Sciences, Jožef Stefan Institute, 1000 Ljubljana, Slovenia; david.heath@ijs.si (D.H.); Ester.Heath@ijs.si (E.H.); 3 Jožef Stefan International Postgraduate School, 1000 Ljubljana, Slovenia; 4 Slovenian Beekeepers’ Association, 1225 Lukovica, Slovenia; andreja.kandolf@czs.si

**Keywords:** neonicotinoids, propolis, SPE, QuEChERS, LC-MS/MS

## Abstract

In this study, a method was developed for the determination of five neonicotinoid pesticides (acetamiprid, clothianidin, imidacloprid, thiacloprid, and thiamethoxam) in propolis. Two sample preparation methods were tested: solid-phase extraction and the quick, easy, cheap, effective, rugged, and safe (QuEChERS) method. The identities of analytes were confirmed using liquid chromatography–tandem mass spectrometry (LC-MS/MS) in the selected reaction monitoring mode. Solid-phase extraction resulted in cleaner extracts; therefore, the SPE-LC-MS/MS method was validated according to the SANTE protocol in triplicate at two spiking levels (10 ng/g and 50 ng/g). The average recoveries of analytes ranged from 61% to 101%, except for clothianidin (10–20%). The LOD ranged from 0.2 ng/g to 4.4 ng/g, whereas the LOQ was in the range of 0.8 ng/g–14.7 ng/g. In order to compensate for the matrix effect, matrix-matched calibration was used. Good accuracy (relative error: 1.9–10.4%) and good linearity (R^2^ > 0.991) were obtained for all compounds. The optimised method was applied to 30 samples: 18 raw propolis and 12 ethanol tinctures. Acetamiprid, imidacloprid, and thiacloprid were detectable in seven samples but were still below the LOQ. This study is the first to report the determination of several neonicotinoid residues in propolis.

## 1. Introduction

Neonicotinoids are the most widely used class of insecticides worldwide [1]. They are commonly used in more than 120 countries on at least 140 different plant species (fruit trees, vegetables, cereals, and indoor plants) to protect them from sucking insects, such as moths and butterflies [2]. They became commercially available by the early 1990s [3].

Neonicotinoid activity is based on their interaction with the central and peripheral nervous system of insects. They have agonist activity on the postsynaptic nicotinic acetylcholine receptor (nAChR), which causes permanent depolarisation of the membrane leading to the blocking of signal transmission [4,5]. Neonicotinoids are considered selectively toxic to insects and have relatively low toxicity to mammals. This assumption has recently been challenged by Kimura-Kuroda et al., who showed that neonicotinoids could affect the mammalian nervous system, especially when it is still developing [6]. Although their impact on mammals could be problematic, they are even more lethal to bees. Because they are systemic pesticides, they can be translocated to all plant tissues, including pollen and nectar, which are the primary food source for bees [7]. Low amounts of these insecticides can cause a lack of orientation and coordination, immune suppression, and general bee inactivity. Lu et al. showed that even sublethal exposure could cause total paralysis and lead to colony collapse disorder (CCD) [8]. For honeybees, nearly all neonicotinoids are toxic. Toxicity is expressed in terms of LD_50_ and is dependent on the neonicotinoid and the type of exposure (oral or contact). LD_50_ values for oral exposure are between 0.0037 µg/bee (imidacloprid) and 17.32 µg/bee (thiacloprid) with an outstandingly high value of 53.3 mg/bee for flonicamid [5]. The LD_50_ values for contact exposure are between 0.0179 µg/bee (imidacloprid) and 38.82 µg/bee (thiacloprid), again with an outstanding value of 51.1 mg/bee for flonicamid [5]. The most toxic are nitro-substituted neonicotinoids (clothianidin, dinotefuran, imidacloprid, nitenpyram, and thiamethoxam), whereas cyano-substituted neonicotinoids (acetamiprid, flonicamid, and thiacloprid) are much less toxic. The toxicity is in the range ng/bee for nitro- and in µg/bee for cyano-substituted neonicotinoids, respectively.

Neonicotinoids have been found in beeswax [9], pollen [10,11], honey [10,12,13,14,15], honey liqueur [16], and even honeybees [9,10,13]. Methods for the determination of neonicotinoids usually consist of two steps—sample preparation and instrumental analysis. For sample preparation, traditional liquid-liquid extraction (LLE) [12,17,18,19], dispersive liquid-liquid microextraction (DLLME) [18,20], solid-phase extraction (SPE) [12,15,21,22], and the quick, easy, cheap, effective, rugged, and safe (QuEChERS) method [9,15,16,23] are used. Neonicotinoids are usually determined by liquid chromatography (LC) with diode array (DAD) [14,24], mass spectrometric (MS) [11,12], or, most commonly, MS/MS detection [9,13,15,16,18]. To date, a limited number of studies have been published on pesticide residues in propolis, such as organophosphorous pesticides [25], organochlorine pesticides [26], and different pesticide residues, including thiacloprid [27]. Propolis is known to be a very challenging matrix for residue analysis as it contains many resins, flavonoids, and phenols and also a high amount of waxes, which, when coextracted, result in significant matrix effects. In order to reduce the matrix effect, sample clean-up is important and cannot be omitted [27].

This study aimed to develop a reliable analytical method for the determination of acetamiprid, clothianidin, imidacloprid, thiacloprid, and thiamethoxam in propolis, validate it, and use it to analyse 30 propolis samples collected from Europe and Canada. For sample clean-up, SPE and QuEChERS were tested and compared in terms of extraction recovery and repeatability. Since only a few studies determine pesticide residues, including neonicotinoids, in propolis, and none that only focuses on neonicotinoids, this article contributes to the database of neonicotinoid contamination.

## 2. Results and Discussion

### 2.1. Method Development and Optimisation

In order to obtain maximum recoveries, selectivity, and repeatability, two extraction methods were tested: solid-phase extraction (SPE) and the quick, easy, cheap, effective, rugged, and safe (QuEChERS) method.

For SPE extraction, samples of propolis tinctures with an adjusted ethanol content (1, 5, 10, and 20%) were prepared (Supplementary Materials Table S1). Recoveries of acetamiprid, imidacloprid, and thiacloprid were not significantly affected by ethanol content in the chosen range. The presence of ethanol negatively affected thiamethoxam recovery at 20% content, and clothianidin had low recoveries below 20% at all ethanol percentages. Therefore, the highest possible percentage of ethanol (10%), giving acceptable recoveries for all of the analytes except clothianidin, was adjusted in the samples. Clothianidin and thiamethoxam are the most polar of the analysed compounds (log*K*_ow_ 0.7 and −0.13, respectively) and are, therefore, most affected by the ethanol content of the sample. Clothianidin, however, is poorly retained on the HLB sorbent even at very low ethanol percentages (1%), while its recovery from the pure aqueous solution was 90%.

Next, two different sample volumes were tested (50 mL and 100 mL—Supplementary Materials Table S2). The largest sample volume passed through the SPE cartridge (HLB, 200 mg) without carry-over or break-through of the analytes was 50 mL. The optimum elution solvent was tested using 4 × 5 mL aliquots each of acetonitrile, ethyl acetate, and methanol. In the case of acetonitrile, approximately 98% of analytes were collected in the first fraction.

For QuEChERS, a modified and an unmodified EN method [28] were tested. The two methods were tested using tinctures in acetonitrile (according to the unmodified EN method), where dried ethanol tincture residues were reconstituted in 5 mL acetonitrile or ethanol (Supplementary Materials Table S3). The best recoveries (95–117%) were obtained for samples with acetonitrile. Due to the complexity of the QuEChERS extracts, the second QuEChERS (clean-up) step was modified by adding 100 mg of C18 sorbent and 100 mg of graphitised carbon black (Supplementary Materials Table S4). Although extraction recoveries were good (85–101%), interfering compounds in the sample clogged the mass spectrometer’s ion source. Additional filtering of the extracts through a 0.45 µm membrane filter (CHROMAFIL Xtra PTFE-45/25) before analysis resulted in the loss of 2–14% of the analytes (Supplementary Materials Table S5).

In order to reduce further the complexity of the matrix, a combination of QuEChERS followed by SPE was performed. Double extraction losses, however, resulted in low recoveries, especially for clothianidin and thiamethoxam (3% and 10%, respectively—Supplementary Materials Table S6). For this reason, SPE was selected as the preferred sample preparation method despite low recovery for clothianidin. Clothianidin is not registered in Slovenia, and since the majority of real samples were of Slovenian origin, we did not expect clothianidin to be present.

### 2.2. Validation of SPE-HPLC-MS/MS Method

Table 1 lists the validation parameters for the SPE-LC-MS/MS method. The SANTE/11945/2015 validation protocol [29] was applied (see Section 3.8. for explanation). The method gives good linearity (R^2^ > 0.99) for all analytes over the selected concentration ranges. The method also provided good LODs and LOQs of 0.2–4.4 ng/g and 0.7–14.7 ng/g, respectively. These LOQs are well below the MRLs of neonicotinoids in honey (50 ng/g for acetamiprid, clothianidin, imidacloprid, and thiamethoxam; 200 ng/g for thiacloprid), whereas for propolis, no MRLs are currently established [30]. The matrix effect was low for polar compounds clothianidin and thiamethoxam but substantial for the other three analytes. Propolis contains a large number of nonpolar substances that could remain in the extract and coelute at longer retention times, thus exhibiting signal suppression only for the analytes with lower polarity. According to the SANTE/11945/2015 protocol [29], mean analyte recovery should be in the range of 70–120% with RSD < 20%. The method also gave satisfactory recoveries for acetamiprid, imidacloprid, and thiacloprid at both spiking levels (91–101%). Lower recoveries were obtained for thiamethoxam (61%) at both spiking levels, but due to good repeatability (RSD: 3–21%), lower recoveries were still acceptable. Clothianidin had very low recoveries (10–20%) but acceptable RSD. Due to low recoveries, its LOD and LOQ were an order of magnitude higher than for other analytes, but LOQ was still below the MRL for honey (50 ng/g). Nevertheless, we plan to develop a separate extraction method for clothianidin in future work. Overall accuracy (expressed as relative error) was good at both spiking levels. However, except for imidacloprid (10.4%), more accurate results were obtained at the lower spiking level (1.9–9.6%) than at the higher spiking level (4.5–10.4%) for all analytes. Improved accuracy could be achieved using an isotopically labelled analogue for each compound.

### 2.3. Uncertainty Determination

The combined uncertainty of the method is between 3.5% and 16.3% at the low concentration level and between 5.4% and 12.8% the high concentration level (Table 2). Except for clothianidin, uncertainty is higher at the low concentration level. The most significant contributors to combined uncertainty are U2 (uncertainty in calibration) and U5 (uncertainty in precision).

### 2.4. Analysis of Samples

**Figure 1:**
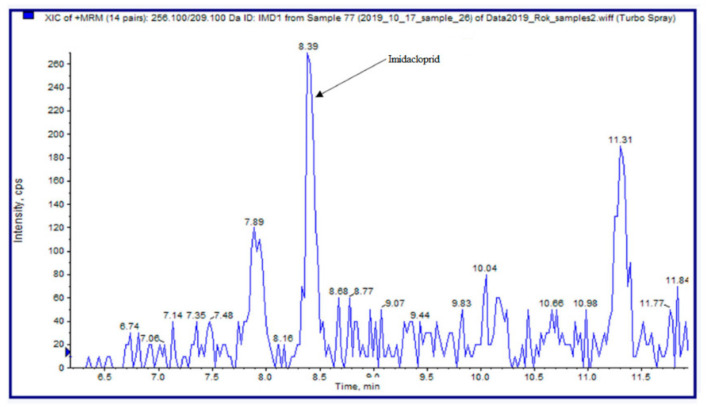
A section of a chromatogram of *m/z* transition for imidacloprid; extract of sample No. 26.

The optimised method was used to analyse 30 propolis samples obtained on the market and from beekeepers (Table 3). In three samples (two Slovenian and one Bulgarian sample), the presence of acetamiprid was confirmed (>LOD); in two samples (one from the Czech Republic and one Serbian sample) the presence of thiacloprid was confirmed; and in two samples (one from the Czech Republic and one Canadian sample), the presence of imidacloprid was confirmed (Figure 1). Concentrations of all detected analytes were between LOD and LOQ. Nevertheless, we calculated the approximate concentrations in positive samples; they are given either in ng/g for raw propolis samples or in μg/L for tinctures in which the actual propolis content was not declared. Low concentrations of analytes in propolis samples can be explained by the composition of propolis, which contains almost 50% of resins from boughs, leaves, and buds of the trees, which are not treated with neonicotinoids, and only on average 5% of pollen, which is the primary source of neonicotinoid contamination [31].

To the best of our knowledge, this is the first study reporting the concentrations of neonicotinoid insecticides in propolis samples. The only published paper on one neonicotinoid (thiacloprid) determination in propolis [27] focused on the clean-up of propolis samples. The accuracy of thiacloprid determination was 81–92% [27], whereas, in the present study, 96–101% was obtained for the same analyte (Table 2). However, no attempt to quantify neonicotinoids in real propolis samples was made [27].

## 3. Materials and Methods

### 3.1. Chemicals and Reagents

Acetamiprid (99.9%), clothianidin (99.1%), clothianidin-d3 (>97%), imidacloprid (99.9%), thiacloprid (99.9%), and thiamethoxam (99.6%) were purchased from Sigma-Aldrich (Steinheim, Germany). Their chemical structure and molar mass are given in Supplementary Materials Table S7. Acetonitrile and methanol (both HPLC grade) were purchased from J. T. Baker (Deventer, The Netherlands). Ethyl acetate and formic acid (97%) were from Sigma-Aldrich (Steinheim, Germany). NH_3_ solution (25%) was from Gram-Mol (Zagreb, Croatia), and absolute ethanol was purchased from Carlo Erba Reagents (Val de Reuil, France). Prior to use, water used was purified using the MILLI-Q purification system (Millipore, Burlington, MA, USA)—MilliQ water (MQW). QuEChERS extraction was performed using kits (No. 5982-5650) and clean-up kits (No. 5982-5056) purchased from Agilent Technologies (Santa Clara, CA, USA). Oasis solid-phase extraction cartridges (HLB cartridges, 200 mg) were from Waters (Milford, MA, USA).

### 3.2. Standard Solutions

Standard stock solutions (1 g/L) were prepared in acetonitrile, and multicomponent working standards (5.5 mg/L) were prepared by appropriate dilution of the stock solution in acetonitrile with 0.1% HCOOH 1:9 (*v/v*). A standard stock solution of the internal standard (IS) clothianidin-d3 (1 g/L) was prepared in acetonitrile. An IS working solution of 3 mg/L was prepared by diluting the working stock solution with acetonitrile.

The multicomponent standards were used for matrix-matched calibration (Supplementary Materials Table S8), solvent-based calibration (Supplementary Materials Table S8), and for spiking propolis samples. Matrix-matched solutions were prepared (LOQ to 75 µg/L) by spiking 500 µL of the blank propolis extracts with the multicomponent standard. An appropriate volume of the standard solution was transferred to a vial, and the solvent removed by evaporation under a gentle stream of nitrogen. The dried residue was then reconstituted with the blank propolis extract (10% tincture in 90% ethanol, Avicenna Herbal Products LTD, Lampeter, UK), in which the absence of neonicotinoids was confirmed by QuEChERS-LC-MS/MS analysis.

Spiked propolis samples were prepared at low and high concentration levels (10 and 50 µg/L, corresponding to 10 and 50 ng/g in raw propolis, respectively) by adding 0.91 µL and 4.55 µL of multicomponent standard and 25 µL of internal standard solutions to the blank propolis sample (5 mL) before extraction. These spiking levels were chosen at two MRLs for neonicotinoid pesticides in honey in the EU since there are no MRLs set for propolis [30]. All standard solutions were hermetically sealed and stored in the dark at 5 °C for up to a month.

### 3.3. Sample Collection and Preparation

Thirty propolis samples (12 tinctures and 18 raw propolis) were collected from different parts of Europe and Canada. Twelve samples were supplied by the Slovenian Beekeeping Association (Čebelarska zveza Slovenije, Lukovica, Slovenia) and 18 by researchers from Slovenia, Europe, and Canada. Propolis tinctures (5 mL) were diluted with MQW (45 mL). Raw propolis samples were dissolved as follows: approximately 0.5 g of sample was ground and mixed with 50 mL of 10% ethanol in MQW. The mixture was shaken on an orbital shaker (IKA KS, 4000 i control) at 300 rpm for 5 h at 50 °C. Samples were then stored in a dark place for five days. All samples were centrifuged at 5000 rpm before extraction.

### 3.4. Solid-Phase Extraction

Solid-phase extraction was optimised using aqueous samples, with approximately 10% of EtOH. Oasis HLB extraction cartridge was conditioned with 5 mL of methanol and washed with 5 mL of MQW. The sample was loaded under a slight vacuum, and the cartridge washed with 10 mL of an NH_3_ aqueous solution (25%) and MeOH (9:1; pH = 11). After the sorbent was dried under vacuum (15 min), the analytes were eluted with 5 mL of acetonitrile. The eluate was then spiked (25 µL) with the internal standard solution and evaporated to dryness under a gentle stream of nitrogen. The residue was afterwards dissolved in 0.5 mL of acetonitrile.

### 3.5. QuEChERS

The QuEChERS method used was based on a modified version of the original EN method [28]. QuEChERS method was optimised for solid samples, so the solvent from the tincture was first evaporated using a rotary evaporator. The dried residue was then dissolved in 10 mL of acetonitrile and transferred into 50 mL centrifuge tube containing 10 mL of MQW and the following salts: MgSO_4_ (6 g), NaCl (1 g), sodium citrate (1 g), and disodium citrate sesquihydrate (0.5 g). The mixture was shaken for 15 min at 300 rpm and then centrifuged for 10 min at 3000 rpm. Six millilitres of supernatant was transferred into 15 mL centrifuge. To this was added 900 mg of anhydrous MgSO_4_ and 150 mg of the primary-secondary amine (PSA) sorbent. The mixture was shaken for 2 min and centrifuged for 5 min at 3000 rpm. The supernatant (1.5 mL) was transferred to an LC vial and analysed by LC-MS/MS.

### 3.6. LC-MS/MS

LC-MS/MS analysis was performed using a Perkin Elmer Series 200 HPLC instrument, coupled to triple quadrupole MS (Applied Biosystems/MDS Sciex 3200 QTRAP, Framingham, MA, USA) with a TurboIonSpray (electrospray ionisation, ESI) source. Analyst 1.6 Software (Applied Biosystems/MDS Analytical Technologies Instruments, Framingham, MA, USA) was used for data acquisition and processing. The mass analyser was used in the selected reaction monitoring (SRM) mode. The separation was achieved using an Agilent C8 column (150 × 4.6 mm, 3.5 μm; Agilent Technologies, Santa Clara, CA, USA). The injection volume was 20 µL, and the flow rate was 0.8 mL/min. The mobile phase was A: 0.1% HCOOH and B: acetonitrile. Gradient elution was used as follows: 0 min, 10% B; 1 min, 10% B; 10 min, 70% B; 10.5 min, 90% B; 12 min, 90% B; 12.1 min, 10% B. Ionisation by ESI in positive mode (4500 V) was used, with ion source at 500 °C. Ion source gases (curtain gas, nebuliser gas, and heater gas) were nitrogen 5.0 at 10, 50, and 60 psi, respectively [15]. Precursor, quantification, and confirmation ions in SRM mode are shown in Table 4.

### 3.7. Matrix-Matched Calibration

In order to compensate for the matrix effect, five-point matrix-matched calibration (MMC) was used. Matrix-matched solutions were prepared as described in Section 3.2., and solvent calibration (SC) was performed to study the matrix effect. The matrix effect was calculated as the slope ratio between MMC and SC calibration: *k*_MMC_/*k*_SC_ (%). A SRM chromatogram of a matrix-matched solution is given in the Supplementary Materials Figure S1. Some peak distortion was observed because the matrix-matched solutions had to be prepared in pure acetonitrile to dissolve the propolis extract.

### 3.8. Method Validation

Validation was performed according to the SANTE/11945/2015 protocol [29], but with only three repetitions instead of five. The SANTE protocol valid at the time of the study was applied although a new protocol (SANTE/12682/2019 [32]) has since entered into force. Validation of SPE-HPLC-MS/MS method was performed at two spiking levels: 10 and 50 µg/L, corresponding to 10 and 50 ng/g of raw propolis, with three parallel determinations at each level. Specificity was determined by analysing the blank sample and matrix-matched solutions. Identification was based on retention time, precursor ion, two product ions, and the ratio between the latter. In order to confirm the analyte identity, all four parameters had to match. Linearity was determined based on matrix-matched calibration (from LOQ to 75 µg/L) and expressed as the coefficient of determination (R^2^). Matrix-matched calibration was also used to determine the LOD and LOQ. The LOD was determined as the concentration at an S/N ratio of 3 and LOQ at S/N = 10. Method (extraction) efficiency was calculated as average recovery and repeatability as RSD of the parallel determinations. Accuracy was expressed as the relative error.

### 3.9. Calculation of Uncertainty

Combined uncertainty was determined according to the procedure described in the EURACHEM/CITAC Guide (2017) for each analyte at two concentration levels (10 and 50 µg/L) [33]. The method consists of four main steps: (a) specification of the measurand, (b) identifying and analysing uncertainty sources, (c) quantifying the uncertainty components, and (d) calculating the combined standard uncertainty.

(a) Specification of the measurand: Measurand was mass concentration (µg/L) of neonicotinoid pesticides in propolis samples.

(b) Identifying and analysing uncertainty sources: The following sources of uncertainty were identified: standard solutions (*U*1), calibration (*U*2), weighing (*U*3), pipetting and dilution (*U*4), and precision (*U*5). The uncertainty associated with differences in the analyte signal in the spiked propolis samples compared to the analyte signal in real propolis samples was considered negligible compared to combined method uncertainty.

(c) Uncertainty components quantification:

- The uncertainty of standard solutions (*U*1): Uncertainty of standard solution consisted of three primary sources: purity of the standard, the mass of the analytical standard, and volume of the standard solution.

The purity of the standard was given on the certificate as 0.9999 ± 0.0001 provided by the manufacturer. In order to calculate the uncertainty, rectangular distribution was assumed, as there was no additional information about the uncertainty.

(1) $$u\left(P\right)=\;\frac{0.0001}{\sqrt{3}}$$

Uncertainty related to the mass of the analytical standard was determined using the data from the accuracy of scales given in the analytical scales certificate.

(2) $$u\left(m\right)=\;0.0001\;\mathrm{mg}$$

Uncertainty of volume of standard solution consisted of three sources:

Calibration uncertainty: It was the result of the value of the uncertainty of the flask and pipettes. The manufacturer gives uncertainty as *V* ± a, without a confidence level of distribution, therefore triangular distribution was assumed.

(3) $$u\left(calibration\right)=\;\frac{a}{\sqrt{6}}$$

Repeatability: It was included in the uncertainty related to the precision of the method. Therefore, it was not calculated separately.

Temperature: Uncertainty related to temperature fluctuation can be calculated from the estimate of the temperature range and the coefficient of the volume expansion of the used solvents:

(4) $$u\left(temperature\right)=\;\frac{V\times ∆\times \alpha_{V}}{\sqrt{3}}$$

where *V* is the volume of standard solution, ∆ is the estimated temperature range (°C) and $\alpha_{V}$ is the solvent expansion coefficient (°C^−1^). The temperature in the laboratory was in the range of 20 ± 0.2 °C, and solvent expansion coefficient of water is 2.1 × 10^−4^ °C^−1^ [34], and that of acetonitrile is 1.4 × 10^−3^ °C^−1^ [35]. Uncertainty due to temperature range was considered negligible.

Therefore, uncertainty related to volume was calculated as:

(5) $$u\left(V\right)=\sqrt{{\left(\frac{u\left(meas.\;flask\;calib.\;\right)}{V}\right)}^{2}+{\left(\frac{u\left(meas.\;pipette\;calib.\;\right)}{V}\right)}^{2}}$$

and standard solution uncertainty as:

(6) $$U_{1}=\sqrt{{\left(\frac{u\left(P\right)\;}{P}\right)}^{2}+{\left(\frac{u\left(m\right)\;}{m}\right)}^{2}\;+\;{\left(\frac{u\left(V\right)\;}{V}\right)}^{2}}$$

- Uncertainty of calibration curves (*U2*): the variance of predicted concentration was calculated as:
  (7) $$var\;\left(x_{pred.}\right)=\frac{S}{B}\;\sqrt{\frac{1}{p}+\frac{1}{n}+\frac{{\left(x_{pred.}-\bar{x}\right)}^{2}}{\sum_{j}{\left(x_{j}-\bar{x}\right)}^{2}}}$$
  where *S* is the residual standard deviation, *B* is the slope of the calibration curve, *p* is the number of measurements to determine *x_pred._*, *n* is the number of measurements for the calibration, *x_pred._* is the determined analyte concentration, $\bar{x}$ is the mean value of the different calibration measurements, and $x_{j}$ is the concentration of the standard solution.

Then uncertainty of the predicted concentration was calculated as:

(8) $$u\left(x_{pred.}\right)=\;\sqrt{var\;\left(x_{pred.}\right)}$$

(9) $$U_{2}=\frac{u(x_{pred.})}{x_{pred.}}$$

- Uncertainty of weighing the sample (*U*3):
  (10) $$U_{3}=\frac{u\left(m\right)}{m}$$
  where *u*(*m*) is the uncertainty defined in the analytical scales certificate provided by the manufacturer (0.1 mg).
- Uncertainty of pipetting and dilution (*U*4) during the extraction process was calculated in the same way as the uncertainty related to volume.
- Uncertainty of precision (*U*5) was calculated based on method precision, determined during the validation of the method. It included method recovery variations, pipetting variations, and variations in the detector signal
  (11) $$U_{5}=\frac{RSD}{\sqrt{n}},$$
  where the *RSD* is the relative standard deviation and *n* is the number of replicates.

(d) Combined uncertainty was calculated as:

(12) $$U=c\times \sqrt{U_{1}{}^{2}+U_{2}{}^{2}+U_{3}{}^{2}+U_{4}{}^{2}+U_{5}{}^{2}}$$

Here *c* is the average concentration of the analyte.

Finally, the expanded uncertainty *U*′ was calculated as:

(13) $$U^{\prime }=k\times U$$

The term *k* is the coverage factor (2) at the confidence level of 95%.

## 4. Conclusions

In this work, an SPE-LC-MS/MS analytical method was developed and optimised for the determination of trace residues of selected neonicotinoids in propolis. To the best of our knowledge, this is the first report on an analytical method for only neonicotinoid insecticides in propolis and the first report on their concentrations in real propolis samples. For sample pretreatment, SPE and QuEChERS extraction methods were compared. Less complex extracts were obtained by SPE extraction at similar recoveries, and therefore, SPE-LC-MS/MS method was validated and applied to the analysis of 30 propolis samples. The analysis showed the presence of acetamiprid, imidacloprid, and thiacloprid in seven samples. Concentrations of all detected analytes were below the LOQ of the method. In conclusion, contamination of propolis with neonicotinoids is low compared to other bee products, such as pollen and honey, which is likely related to the origin and constituents of the propolis.

## Figures and Tables

**Table 1:** Validation results of the SPE-LC-MS/MS method.

Analyte	Linearity	LOD (ng/g)	LOQ (ng/g)	Matrix Effect (*k*_MMC_/*k*_SC_)	Recovery (%)		Accuracy *E*_R_ (%)		Repeatability RSD (%)	
					L	H	L	H	L	H
Acetamiprid	0.994	0.2	0.7	0.49	95	91	7.1	8.8	27	8
Clothianidin	0.991	4.4	14.7	0.99	10	20	2.8	9.5	5	6
Imidacloprid	0.996	0.7	2.2	0.54	99	95	9.6	4.5	29	12
Thiacloprid	0.991	0.2	0.8	0.3	101	96	1.9	10.4	20	13
Thiamethoxam	0.994	0.3	1.0	0.81	61	61	7.2	8.3	21	3

*E*_R_—relative error. L—low concentration level (10 ng/g). H—high concentration level (50 ng/g). *k*_MMC_—slope for calibration with matrix-matched solutions. *k*_SC_—slope for calibration with multicomponent standard working solutions (solvent calibration).

**Table 2:** Uncertainty calculated at two concentration levels for SPE-LC-MS/MS method.

Analyte	U1		U2		U3		U4		U5		Rel.U’	
	L	H	L	H	L	H	L	H	L	H	L	H
Acetamiprid	0.0060	0.0060	0.0581	0.0164	0.0002	0.0002	0.0024	0.0024	0.0410	0.0508	0.1429	0.1076
Clothianidin	0.0060	0.0060	0.0032	0.0008	0.0002	0.0002	0.0024	0.0024	0.0162	0.0548	0.0354	0.1105
Imidacloprid	0.0060	0.0060	0.0038	0.0009	0.0002	0.0002	0.0024	0.0024	0.0554	0.0260	0.1119	0.0536
Thiacloprid	0.0060	0.0060	0.0805	0.0215	0.0002	0.0002	0.0024	0.0024	0.0110	0.0600	0.1630	0.1282
Thiamethoxam	0.0060	0.0060	0.0556	0.0149	0.0002	0.0002	0.0024	0.0024	0.0416	0.0479	0.1395	0.1012

U1—uncertainty of standard solutions. U2—uncertainty of calibration curves. U3—uncertainty of weighing. U4—uncertainty of pipetting and dilution operations. U5—uncertainty of precision. Rel. U′—relative expanded uncertainty. L—low concentration level (10 ng/g). H—high concentration level (50 ng/g).

**Table 3:** Characteristics of analysed propolis samples and concentration of neonicotinoids.

Sample Number	Raw/Tincture	Origin	Concentration
1	raw	Slovenia	ND
2	raw	Slovenia	ND
3	raw	Slovenia	ND
4	raw	Slovenia	ND
5 ^1^	raw	Slovenia	ND
6	raw	Slovenia	Acetamiprid (0.35 ng/g) ^2^
7	raw	Slovenia	ND
8	raw	Slovenia	ND
9	raw	Slovenia	ND
10	raw	Slovenia	ND
11 ^1^	raw	Slovenia	Acetamiprid (0.41 ng/g) ^2^
12	raw	Slovenia	ND
13	raw	Slovenia	ND
14	raw	Slovenia	ND
15	tincture	Bulgaria	Acetamiprid (0.39 μg/L) ^2^
16	tincture	Czech Republic	Thiacloprid (0.45 μg/L) ^2^
17 ^1^	tincture	Czech Republic	Imidacloprid (0.97 μg/L) ^2^
18	tincture	Serbia	ND
19	tincture	Slovenia	ND
20	raw	Slovenia	ND
21	tincture	Serbia	Thiacloprid (0.39 μg/L) ^2^
22 ^1^	tincture	Slovenia	ND
23	raw	Slovenia	ND
24	tincture	Belgium	ND
25	tincture	Greece	ND
26	tincture	Canada	Imidacloprid (0.99 μg/L) ^2^
27	tincture	Italy	ND
28	tincture	Croatia	ND
29	raw	Slovenia	ND
30	raw	Slovenia	ND

^1^ Sample only analysed in one parallel due to the low quantity. ^2^ Estimated concentrations (i.e., they are below LOQ). ND—not detected (below LOD).

**Table 4:** Precursor, quantification (*), and confirmation ions used in the selected reaction monitoring (SRM) mode.

Analyte	Precursor Ion (*m/z*)	Product Ion (*m/z*)	Declustering Potential (V)	Collision Energy (V)
Acetamiprid	223.2	126.0 *	45	25
		90.3	45	44
Clothianidin	250.2	169.3 *	32	19
		131.9	32	22
Imidacloprid	256.1	209.1 *	40	20
		175.1	40	26
Thiacloprid	253.1	126.0 *	50	27
		186.1	50	18
Thiamethoxam	291.1	211.2 *	31	16
		181.2	31	30

## Supplementary Materials

The following are available online, Table S1: Recoveries and repeatability of solid-phase extraction for samples with 20, 10, 5, and 1% EtOH. Table S2: Recoveries and repeatability of solid-phase extraction of 50 and 100 mL of the sample with 10% EtOH. Table S3: Recoveries and repeatability of QuEChERS extraction from acetonitrile and ethanol. Table S4: Recoveries and repeatability of QuEChERS extraction with the addition of graphitised carbon black and C18 in dSPE step. Table S5: Recovery of analytes after filtering through 0.45 µm membrane filter (CHROMAFIL Xtra PTFE-45/25). Table S6: Recoveries and repeatability of combined QuEChERS (first step) and solid-phase extraction (second step) methods. Table S7: Name, chemical structure and molecular weight of the analytes. Table S8: List of matrix-matched (used for MMC) and multicomponent standard working solutions (used for SC). Figure S1a: SRM chromatogram of matrix-matched calibration solution (50 µg/L of each analyte). All *m/z* are displayed (except for clothianidin). Figure S1b: SRM chromatogram of matrix-matched calibration solution (50 µg/L of each analyte): clothianidin *m/z* are displayed.
