# Supplementary material for: Determination of Neonicotinoid Pesticides in Propolis with Liquid Chromatography Coupled to Tandem Mass Spectrometry
Source: Molecules. 2020 Dec 11;25(24):5870. doi: 10.3390/molecules25245870 (PMC7764281; doi:10.3390/molecules25245870)

Determination of neonicotinoid pesticides in propolis with liquid chromatography coupled to tandem mass spectrometry

Supplementary Material

Rok Tomšič[a], David Heath[b], Ester Heath[b,c], Jernej Markelj[a], Andreja Kandolf Borovšak[d], Helena Prosen[a,*]

a Faculty of Chemistry and Chemical Technology, University of Ljubljana, Ljubljana, Slovenia

b Department of Environmental Sciences, Jožef Stefan Institute, Ljubljana, Slovenia

c Jožef Stefan International Postgraduate School, Ljubljana, Slovenia

d Slovenian Beekeepers' Association, Lukovica, Slovenia

* Correspondence: helena.prosen@fkkt.uni-lj.si; Tel.: +386-1-479-8556

Table S1: Recoveries and repeatability of solid phase extraction of samples with 20, 10, 5 and 1% EtOH.

	20% EtOH		10% EtOH		5% EtOH		1% EtOH	
	**Recovery [%]**	**Repeatability RSD [%]**	**Recovery [%]**	**Repeatability RSD [%]**	**Recovery [%]**	**Repeatability RSD [%]**	**Recovery [%]**	**Repeatability RSD [%]**
Acetamiprid	100	10	82	27	94	10	83	9
Clothianidin	10	3	11	2	18	3	14	3
Imidacloprid	88	11	89	24	82	6	73	9
Thiacloprid	98	8	83	19	91	8	79	17
Thiamethoxam	30	4	66	25	57	5	57	6

Table S2: Recoveries and repeatability of solid phase extraction of 50 and 100 mL of a sample with 10% EtOH.

	50 mL		100 mL	
	**Recovery [%]**	**Repeatability RSD [%]**	**Recovery [%]**	**Repeatability RSD [%]**
Acetamiprid	77	6	36	3
Clothianidin	15	2	0	0
Imidacloprid	76	5	1	0
Thiacloprid	80	5	94	6
Thiamethoxam	53	3	0	0

Table S3: Recoveries and repeatability of QuEChERS extraction from acetonitrile and ethanol.

	ACN		EtOH	
	**Recovery [%]**	**Repeatability RSD [%]**	**Recovery [%]**	**Repeatability RSD [%]**
Acetamiprid	111	17	88	31
Clothianidin	95	15	106	25
Imidacloprid	95	12	90	33
Thiacloprid	117	18	102	32
Thiamethoxam	98	23	82	32

Table S4: Recoveries and repeatability of QuEChERS extraction with the addition of graphitized carbon black and C_{18} in dSPE step.

	**Recovery [%]**	**Repeatability RSD [%]**
Acetamiprid	95	16
Clothianidin	91	7
Imidacloprid	101	6
Thiacloprid	100	11
Thiamethoxam	85	16

Table S5: Recovery of analytes after filtering through 0.45 µm membrane filter (CHROMAFIL Xtra PTFE-45/25).

	**Recovery [%]**	**Repeatability RSD [%]**
Acetamiprid	98	10
Clothianidin	86	11
Imidacloprid	97	15
Thiacloprid	95	14
Thiamethoxam	97	13

Table S6: Recoveries and repeatability of combined QuEChERS (first step) and solid phase extraction (second step) methods.

	**Recovery [%]**	**Repeatability RSD [%]**
Acetamiprid	61	12
Clothianidin	3	1
Imidacloprid	63	5
Thiacloprid	109	20
Thiamethoxam	10	2

Table S7: Name, chemical structure and molecular weight of the analytes.

Name	Chemical structure	MW [g/mol]
Acetamiprid	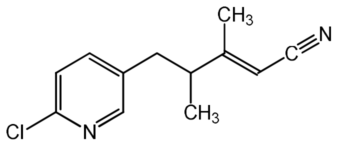	222.7
Clothianidin	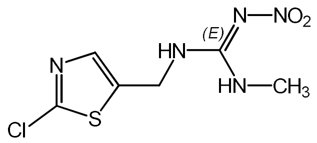	249.7
Imidacloprid	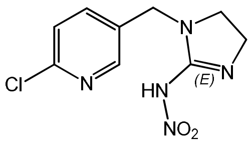	255.7
Thiacloprid	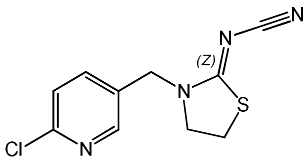	252.7
Thiamethoxam	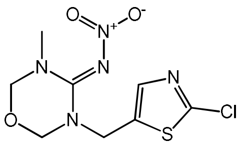	291.7

Reference: https://pubchem.ncbi.nlm.nih.gov/

Table S8: List of matrix-matched (used for MMC) and multicomponent standard working solutions (used for SC).

SC [µg/L]	MMC [µg/L]
0.20[a]	0.20[b]
0.99	1.00[c]
2.50	2.50
12.35	12.51
24.97	25.01
49.95	50.03
74.65	75.03

[a] Only for acetamiprid and thiacloprid.
[b] Only for acetamiprid
[c] Only for acetamiprid, thiacloprid and thiamethoxam

Figure S1: a) SRM chromatogram of matrix-matched calibration solution (50 µg/L of each analyte). All *m*/*z* are displayed (except for clothianidin).

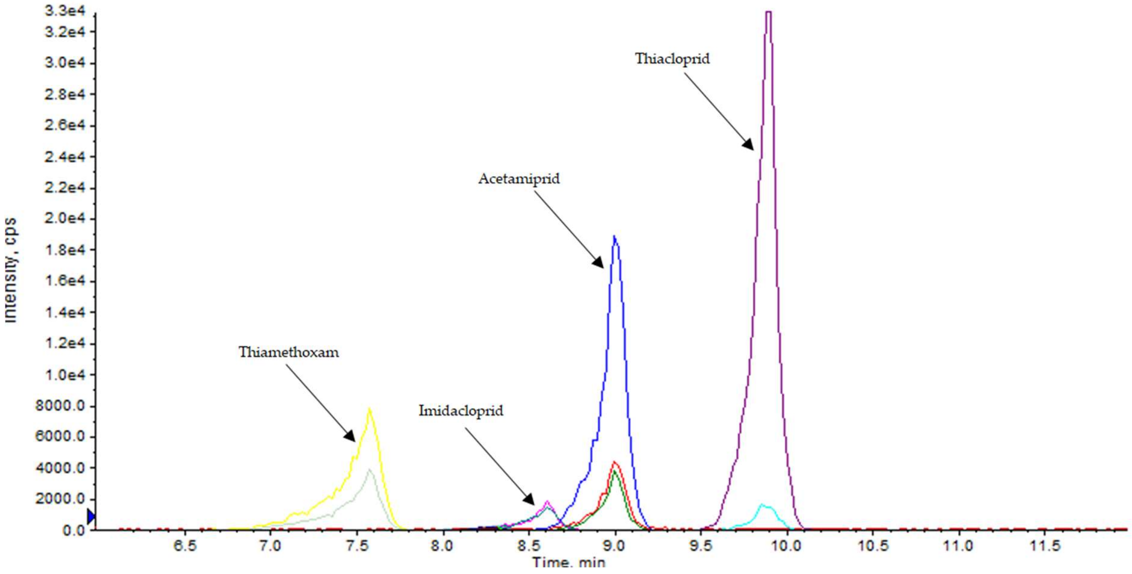

Figure S1: b) SRM chromatogram of matrix-matched calibration solution (50 µg/L of each analyte): clothianidin *m*/*z* are displayed.

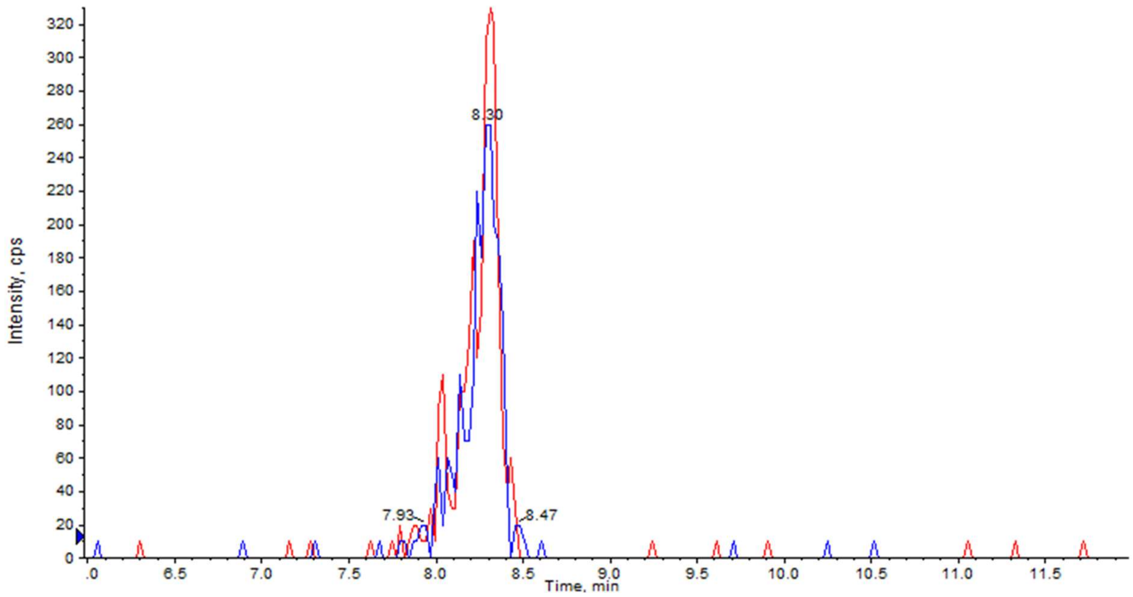

Supplement: Supplementary file 1 [file molecules-25-05870-s001.pdf]
